# Dual-Band High-Throughput and High-Contrast All-Optical Topology Logic Gates

**DOI:** 10.3390/mi15121492

**Published:** 2024-12-13

**Authors:** Jinying Zhang, Yulin Si, Yexiaotong Zhang, Bingnan Wang, Xinye Wang

**Affiliations:** 1 Beijing Key Lab for Precision Optoelectronic Measurement Instrument and Technology, School of Optics and Photonics, Beijing Institute of Technology, Beijing 100081, China; 3120210633@bit.edu.cn (Y.S.); 3120220661@bit.edu.cn (Y.Z.); 3120215361@bit.edu.cn (B.W.); 3120205353@bit.edu.cn (X.W.); 2 Yangtze Delta Region Academy of Beijing Institute of Technology, Jiaxing 314001, China

**Keywords:** topological photonic crystals, all-optical logic gates, dual-band, high-throughput

## Abstract

Optical computing offers advantages such as high bandwidth and low loss, playing a crucial role in signal processing, communication, and sensing applications. Traditional optical logic gates, based on nonlinear fibers and optical amplifiers, suffer from poor robustness and large footprints, hindering their on-chip integration. All-optical logic gates based on topological photonic crystals have emerged as a promising approach for developing robust and monolithic optical computing systems. Expanding topological photonic crystal logic gates from a single operating band to dual bands can achieve high throughput, significantly enhancing parallel computing capabilities. This study integrates the topological protection offered by valley photonic crystals with linear interference effects to design and implement seven optical computing logic gates on a silicon substrate. These gates, based on dual-band valley photonic crystal topological protection, include OR, XOR, NOT, NAND, NOR, and AND. The robustness of the implemented OR logic gates was verified in the presence of boundary defects. The results demonstrate that multi-band parallel computing all-optical logic gates can be achieved using topological photonic crystals, and these gates exhibit high robustness. The all-optical logic gates designed in this study hold significant potential for future applications in optical signal processing, optical communication, optical sensing, and other related areas.

## 1. Introduction

All-optical computing, characterized by advantages such as high bandwidth and low loss, plays a crucial role in diverse applications, including signal processing, communication, and sensing [1,2,3,4]. Optical logic gates constitute the fundamental building blocks of all-optical computing. Traditional implementations of optical logic gates typically rely on nonlinear fiber couplers [5,6] and optical amplifiers [7,8,9]. Nonlinear fiber couplers leverage nonlinear effects, utilizing waveguides composed of materials with intensity-dependent refractive indices. The operational state of the coupler is controlled by modulating the input optical signal intensity to achieve the desired logic gate functionality. However, such optical logic gates are characterized by their bulky size, which hinders monolithic integration. Moreover, their susceptibility to environmental interference leads to poor robustness [10]. Optical amplifiers exhibit high nonlinearity and offer ease of integration. By utilizing the input signal light to induce changes in the internal carrier concentration, the refractive index of the active region is modulated, resulting in variations in both phase and gain. These variations ultimately produce the desired logic functions. However, these logic gates are susceptible to self-radiation during operation, potentially impacting their transmission speed and response time.

Driven by the increasing demand for miniaturization and lightweight all-optical computing systems, compact optical logic devices have attracted significant research interest. For example, Cuicui Lu et al. fabricated a ferroelectric hybrid plasmonic waveguide to realize an all-optical OR gate [11]. Despite its compact footprint, this device exhibits significant optical loss. Similarly, Meng Xiong et al. demonstrated an all-optical AND gate based on a single silicon microring resonator [12]. While capable of performing the AND logic function, the device suffers from wavelength conversion between the input and output signals, hindering cascaded logic operations and impeding large-scale computation. Yulan Fu et al. theoretically demonstrated high-contrast and low-power all-optical logic gates in a two-dimensional silicon photonic crystal, leveraging beam interference effects [13]. Despite their ability to implement various logic gates, photonic crystals [5,13,14,15,16,17,18] require stringent fabrication precision, remain susceptible to perturbations, and exhibit limited robustness. Topological photonic crystals, on the other hand, offer exceptionally robust light transmission capabilities. These crystals exhibit topologically protected, highly efficient unidirectional transmission, even in the presence of certain disorders along the transmission path [19,20,21]. Consequently, they have emerged as a prominent research area in recent years.

In 2020, a theoretical proposal for all-optical logic gates based on topologically protected two-dimensional silicon photonic crystals was presented [22]. Leveraging the topologically protected edge states within valley photonic crystals, Minghao Chao and colleagues demonstrated novel optical XOR and OR logic gates in 2021 [23]. Expanding upon the theoretical framework of topologically protected all-optical logic gates, Professor Xiangdong Zhang’s group [10] designed and experimentally validated seven types of topologically protected all-optical logic gates using silicon valley photonic crystals in 2023. This achievement represents a significant advancement in the field. However, these optical logic gates rely on single-band topological photonic crystals, which limits further enhancements in computational capabilities. Moreover, the reliance on ABC-type topological valley photonic crystals for establishing robust optical transmission paths restricts the potential for the miniaturization of optical logic gates. This study proposes and designs all-optical logic gates based on dual-band AC-type topological photonic crystals. By integrating the topological protection of valley photonic crystals with linear interference effects, the proposed approach realizes seven types of topologically protected all-optical logic gates on a silicon substrate, including OR, XOR, NOT, NAND, NOR, XNOR, and AND. This approach maintains design flexibility and transmission robustness while enabling the expansion of operating bandwidth and a further reduction in device size. Consequently, it holds promise for the development of all-optical computing chips with enhanced computing power and higher integration density. 

## 2. Structure and Band Structure Analysis of Topological Valley Photonic Crystals

**Figure 1:**
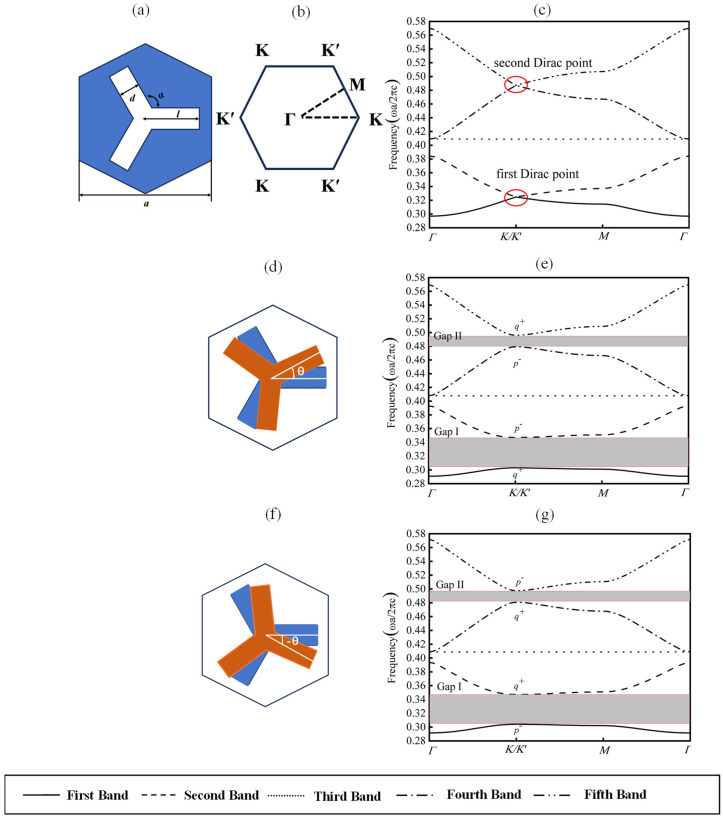
Schematic of two-dimensional photonic crystal structure and its band structures. (**a**) Schematic of photonic crystal structure with *θ* = 0°; (**b**) view of the first Brillouin zone of a triangular lattice, dashed triangle marked *k*-path for band calculation; (**c**) photonic crystal band structure with *θ* = 0°; (**d**) schematic of photonic crystal rotation structure at *θ* = 5° (blue represents scattered body at *θ* = 0°, red represents scatterer at *θ* = 5°); (**e**) photonic crystal band structure with *θ* = 5°; (**f**) schematic of photonic crystal rotation structure at *θ* = −5° (blue represents scattered body at *θ* = 0°, red represents scatterer at *θ* = −5°); (**e**) photonic crystal band structure with *θ* = 5°; the curves in (**e**,**g**) represent the first band, second band, third band, fourth band, and fifth band in sequence from top to bottom.

Figure 1a illustrates the designed unit cell of a triangular lattice composed of Y-shaped scatterers with a lattice constant of *a* = 0.52 μm. Each scatterer (represented by the white region) comprises three rectangles with lengths of *l* = 0.21 μm, widths of *d* = 0.08 μm, and an angle of *α* = 120° between adjacent rectangles. The background material (depicted in blue) consists of a silicon substrate with a dielectric constant of 11.7. The scatterer is made of metal material, and a perfect electrical conductor is used instead in the simulation. Figure 1b shows the rotation of the scatterers within the structure by an angle, *θ*. Setting the rotation angle, *θ =* 0°, and scanning along the dashed triangular path *Γ*-*K*-*M*-*Γ* within the Brillouin zone (Figure 1c) reveals two Dirac cones at the K (K′) points in the band diagram (Figure 1d). These Dirac cones, distinguished by their distinct frequencies, are protected by the *C*_3*v*_ point group symmetry. This photonic crystal configuration is designated as type B. However, rotating the scatterers by *θ* = 5° (counterclockwise) or *θ* = −5° (clockwise) breaks the spatial inversion symmetry, reducing the point group symmetry to *C*_3_ and lifting the two-fold degeneracy. It is important to clarify that the introduction of topological phase transition does not depend on the choice of the value, meaning that this angle can be freely selected. Consequently, the bandgaps of the two Dirac cones open, forming Gap I and Gap II, as shown in Figure 1e,f. Gap I corresponds to a frequency range of 174.73 THz to 200.11 THz (wavelengths of 1499.18 nm to 1716.93 nm), while Gap II corresponds to a frequency range of 276.36 THz to 285.99 THz (wavelengths of 1085.54 nm to 1106.12 nm). Photonic crystals with rotation angles of *θ* = 5° and *θ* = −5° are designated as type A and type C, respectively.

The Berry curvature of the bands below Gap I and Gap II can be determined utilizing the k·p perturbation method, where k represents the crystal momentum and p denotes the momentum operator. The explicit expression derived from this method is as follows:

$$\Omega \left(\delta k\right)=m_{i}v_{Di}/[2{\left(\delta k^{2}+{m_{i}}^{2}+{v_{Di}}^{2}\right)}^{\frac{3}{2}}]$$

In the aforementioned expression, $v_{Di}$ denotes the Dirac cone dispersion velocity at a zero-rotation angle (θ=0°). Here, θ represents the rotation angle, signifying the orientational degree of freedom. The term δk corresponds to the momentum deviation from the K point within the Brillouin zone. The effective mass, mi, is defined as $m_{i}=\left(\omega_{q+}^{i}-\omega_{q-}^{i}\right)/\left(2v_{Di}^{2}\right)$, where $\omega_{q+}^{i}$ and $\omega_{q-}^{i}$ represent the band frequencies for counterclockwise and clockwise energy flow, respectively.

Utilizing the k·p perturbation method [24,25,26], the valley Chern numbers at the K valley for Gap I are calculated to be 1/2 and −1/2 for type A and type C photonic crystals, respectively, resulting in a valley Chern number change of 1. Similarly, for Gap II, the valley Chern numbers at the K valley are calculated to be −1/2 and 1/2 for type A and type C photonic crystals, respectively, resulting in a valley Chern number change of −1. By combining type A and type C photonic crystals, which exhibit opposite valley Chern numbers, a boundary supporting the propagation of topological valley edge states can be established.

**Figure 2:**
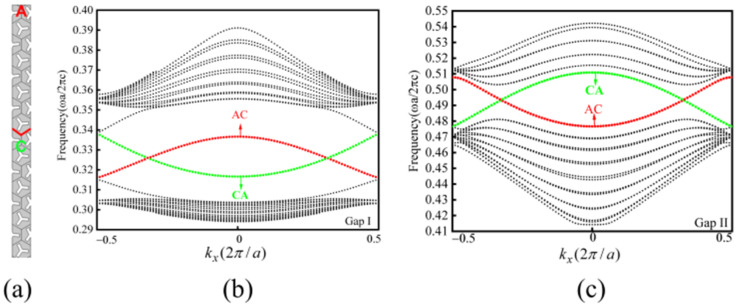
Schematic of AC-type supercell structure with zigzag-type boundary and its band structure. (**a**) Schematic diagram of AC-type supercell structure; (**b**) the band structure of the AC-type supercell structure in Gap I; (**c**) the band structure of the AC-type supercell structure in Gap II. Dashed lines represent both the intrinsic bulk and edge states of the photonic crystal. Refer to the main text for details on their differentiation based on energy and momentum.

In this study, a supercell structure featuring a zigzag-type boundary is constructed by vertically stacking type A and type C photonic crystals, as depicted in Figure 2a. The band structures of the first Brillouin zone for this supercell structure, specifically within Gap I and Gap II, are calculated using finite element simulations, as illustrated in Figure 2b and Figure 2c, respectively. The two bands appearing within the bandgaps represent the topologically protected valley edge states supported by the AC-type and CA-type boundaries.

## 3. Structure Design and Functional Implementation of All-Optical Logic Gates

**Figure 3:**
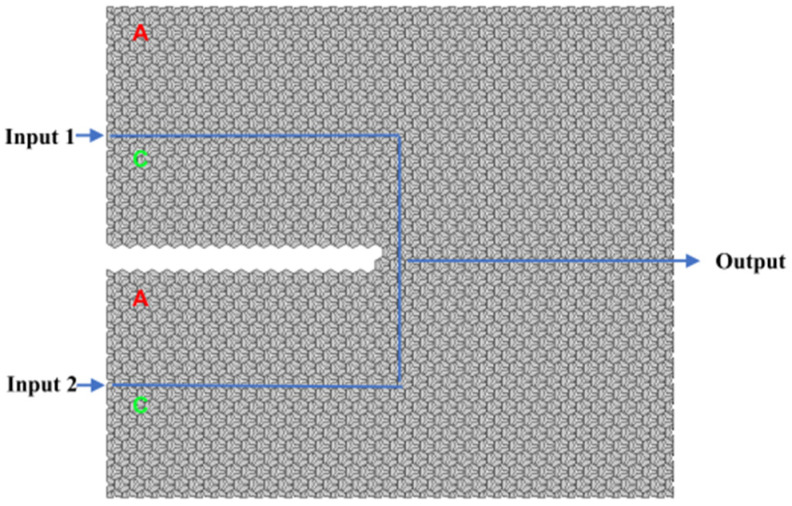
Design diagram of “OR” and “XOR” gate.

By leveraging the aforementioned valley topological photonic crystals in conjunction with linear interference effects, robust all-optical logic gates can be implemented. Versatile logic functionalities can be achieved by judiciously selecting and controlling the input ports. This study utilizes AC-type and CA-type edge states to construct topologically protected OR and XOR gates, as depicted in the schematic diagrams of Figure 3. The topological boundary is designed in a “ψ” shape, incorporating two input ports (input port 1 and input port 2) and a single output port. To ensure consistency, a plane wave excitation with a frequency of 193.54 THz (1550 nm) is applied to the input ports, and scattering boundary conditions are imposed on all surrounding boundaries. When both input ports are activated, the two logical input signals propagate towards the output port and undergo interference. Therefore, by manipulating the phase of the optical excitation signals at the input ports, the designed optical computing logic gates can achieve optical signal interference at the output port, resulting in either constructive or destructive interference to perform optical logic operations.

**Figure 4:**
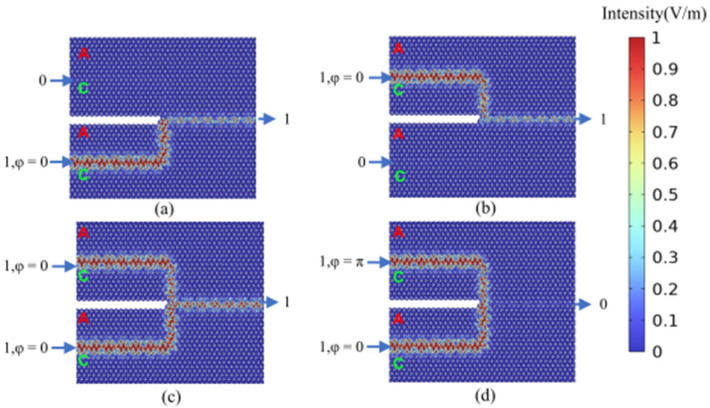
The calculation results of the light field distribution in the logical states of “OR” gate and “XOR” gate. (**a**) “OR/XOR” gate logic input state 01, logic output state 1 (OR_01(XOR_01)); (**b**) “OR/XOR” gate logic input state 10, logic output state 1 (OR_10(XOR_10)); (**c**) “OR” gate logic input state 11, logic output state 1 (OR_11); (**d**) “XOR” gate logic input state 11, logic output state 0 (XOR_11). The color scale represents the magnitude of the electric field (V/m) obtained directly from simulations.

By setting the phase difference between the two input ports to Δ*φ* = 0 (Δ*φ* = π), linear constructive (destructive) interference is generated at the output port, corresponding to an output logic state of 1 (0), respectively. For both OR and XOR gates, when both input port 1 and input port 2 are deactivated (logical input state 00), the field intensity at the output port is zero, indicating an output logic state of 0. As illustrated in Figure 4a, when input port 1 is deactivated and input port 2 is activated (logical input state 01), the output amplitude at the output port is non-zero, representing an output logic state of 1. This behavior aligns with the expected output for both the (01) OR logic and the (01) XOR logic. Similarly, as shown in Figure 4b, when input port 1 is activated and input port 2 is deactivated (logical input state 10), the output amplitude at the output port is non-zero, signifying an output logic state of 1. This aligns with the expected output for both the (10) OR logic and the (10) XOR logic. As depicted in Figure 4c, both input port 1 and input port 2 are activated, with their phase difference set to Δ*φ* = 0. This results in a non-zero amplitude at the output port, representing an output logic state of 1, which aligns with the expected output for the (11) OR logic. Similarly, as illustrated in Figure 4d, both input port 1 and input port 2 are activated (logical input state 11). Setting their phase difference to Δ*φ* = π results in an output amplitude approaching 0, indicating an output logic state of 0 and confirming the (11) XOR logic functionality. The calculated results for all four logic states of the OR and XOR gates are consistent with their respective truth tables, verifying the successful implementation of the desired OR and XOR logic operations. This functionality can be further validated through numerical analysis. The efficiency of logic gates is commonly assessed by calculating the extinction ratio (also known as the contrast ratio), defined as 10log(I_1_/I_0_). Here, I_1_ and I_0_ represent the output intensities corresponding to logic states 1 and 0, respectively. The designed OR and XOR gates exhibit a contrast ratio of 37.16 dB, demonstrating excellent logic gate performance.

**Figure 5:**
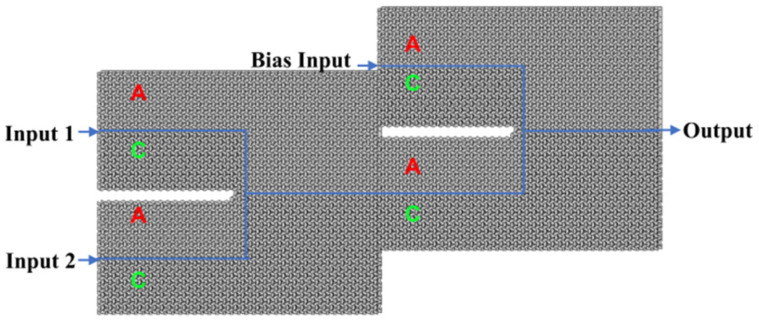
Design diagram of “NOT”, “NAND”, “NOR”, and “XNOR” gates.

More complex optical computing logic gates can be implemented by cascading two or three OR and XOR logic units. Figure 5 illustrates the schematic diagrams for the NOT, NAND, NOR, and XNOR gates. These gates are constructed by cascading two logic gates and incorporating three input ports (input port 1, input port 2, and a bias light input port) as well as a single output port.

**Figure 6:**
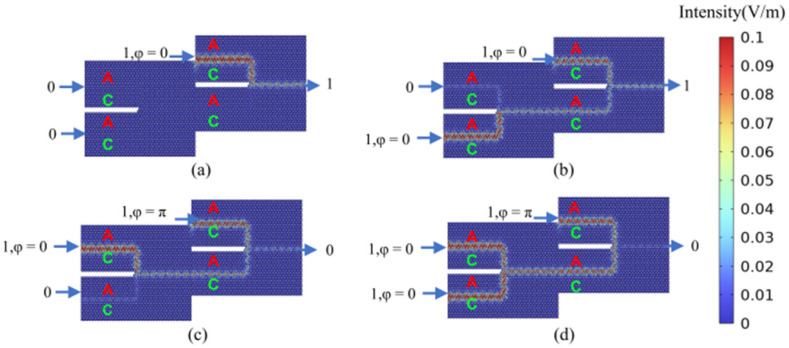
Calculation results of the light field distribution in the logic state of the “NOT” gate. (**a**) “NOT” gate logic input state 00, logic output state 1 (NOT_00); (**b**) “NOT” gate logic input state 01, logic output state 1 (NOT_01); (**c**) “NOT” gate logic input state 10, logic output state (0NOT_10); (**d**) “NOT” gate logic input state 11, logic output state 0 (NOT_11). The color scale represents the magnitude of the electric field, and the scale is adjusted to best visualize the field distribution.

To implement the NOT gate, which outputs the logical negation of the signal at input port 1, the phase difference between input port 1 and input port 2 is maintained at Δ*φ* = 0, while the bias light input is activated. The NOT gate functionality is achieved by controlling the phase of the bias light signal. As illustrated in Figure 6a,b, when the logical input states at input port 1 and input port 2 are 00 or 01, the phase difference between the bias light signal and the signals at the input ports is set to Δ*φ* = 0. Consequently, the output optical signal amplitudes at the output port are non-zero, representing an output logic state of 1. Similarly, as depicted in Figure 6c,d, when the logical input states at input port 1 and input port 2 are 10 or 11, the phase difference between the bias light signal and the signals at the input ports is set to Δ*φ* = π. This results in zero output optical signal amplitudes at the output port, corresponding to an output logic state of 0. Under these operating conditions, the NOT gate achieves a contrast ratio of 24.91 dB. The intensities presented in the figures represent the magnitudes of the electric fields obtained directly from our simulations. The scales may vary between figures to best visualize the field distributions in each specific case. These magnitudes of electric field can be used to analyze the nonlinear item when other optical nonlinear materials are taken into consideration.

**Figure 7:**
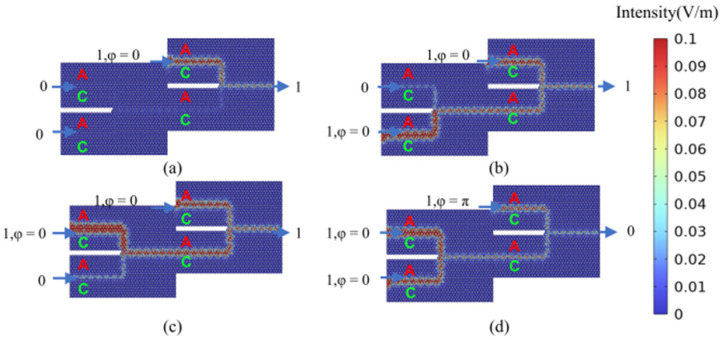
The calculation result of the optical field distribution in the “NAND” gate logic state. (**a**) “NAND” gate logic input state 00, the logic output state 1 (NAND_00); (**b**) “NAND” gate logic input state 01, logic output state 1 (NAND_01); (**c**) “NAND” gate logic input state 10, logic output state 1 (NAND_10); (**d**) “NAND” gate logic input state 11, logic output state 0 (NAND_11).

Other optical computing logic gates can be implemented in a similar manner. A NAND gate (the logical negation of an AND gate applied to input port 1 and input port 2) can be realized by maintaining the phase difference between input port 1 and input port 2 at Δ*φ* = 0 and activating the bias light. The NAND gate logic function is achieved through the control of the bias light signal’s phase. Figure 7a–c illustrate the cases where the logical input states of input port 1 and input port 2 are 00, 01, or 10. In these scenarios, the phase difference between the polarized light signal and the input ports is set to Δ*φ* = 0. This results in non-zero output optical signal amplitudes at the output port, which corresponds to an output logic state of 1. Figure 7d depicts the case where both input port 1 and input port 2 have logical input states of 11. In this instance, the phase difference between the polarized light signal and the input ports is set to Δ*φ* = π. Consequently, the output optical signal amplitude at the output port is zero, indicating an output logic state of 0. Under these operational conditions, the NAND gate exhibits a contrast ratio of 27.96 dB.

**Figure 8:**
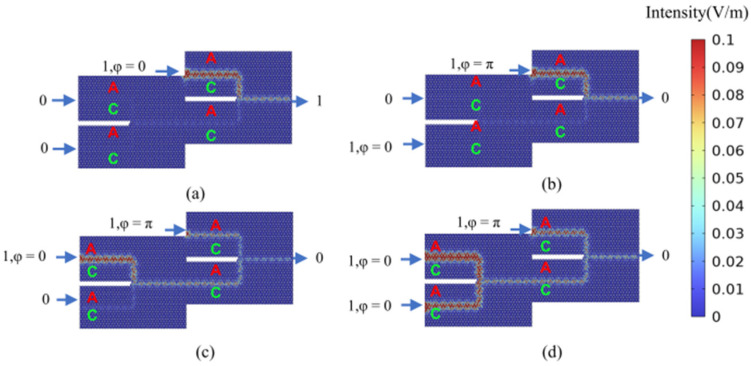
The calculation result of the optical field distribution in the “NOR” gate logic state. (**a**) “NOR” gate logic input state 00, the logic output state 1 (NOR_00); (**b**) “NOR” gate logic input state 01, logic output state 0 (NOR_01); (**c**) “NOR” gate logic input state 10, logic output state 0 (NOR_10); (**d**) “NOR” gate logic input state 11, logic output state 0 (NOR_11).

Analogously, a NOR gate (the logical negation of an OR gate applied to input port 1 and input port 2) can be realized by maintaining the phase difference between input port 1 and input port 2 at Δ*φ* = 0 and activating the bias light. The NOR gate logic function is achieved by controlling the phase of the bias light signal. Figure 8a illustrates the case where both input port 1 and input port 2 have logical input states of 00. In this scenario, the phase difference between the polarized light signal and the input ports is set to Δ*φ* = 0. This results in a non-zero output optical signal amplitude at the output port, which corresponds to an output logic state of 1. Figure 8b–d depict the cases where the logical input states of input port 1 and input port 2 are 01, 10, or 11. In these instances, the phase difference between the polarized light signal and the input ports is set to Δ*φ* = π. Consequently, the output optical signal amplitudes at the output port are reduced to zero, indicating an output logic state of 0. Under these operational conditions, the NOR gate exhibits a contrast ratio of 20.57 dB.

**Figure 9:**
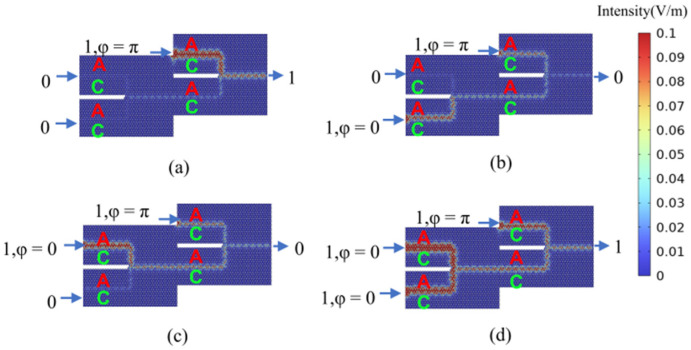
Calculation results of optical field distribution in “XNOR” gate logic state. (**a**) “XNOR” gate logic input state 00, logic output state 1 (XNOR_00); (**b**) “XNOR” gate logic input state 01, logic output state 0 (XNOR_01); (**c**) “XNOR” gate logic input state 10, logic output state 0 (XNOR_10); (**d**) “XNOR” gate logic input state 11, logic output state 1 (XNOR_11).

It is important to note that for the XNOR gate (the logical negation of an XOR gate applied to input port 1 and input port 2), the phase difference between input port 1 and input port 2 is maintained at Δ*φ* = 0, with the bias light activated. The XNOR gate logic function is achieved by controlling the phase difference between the bias light signal and the input ports, setting it to Δ*φ* = π, as illustrated in Figure 9a–d. Under this configuration, the XNOR gate exhibits a contrast ratio of 20.49 dB.

**Figure 10:**
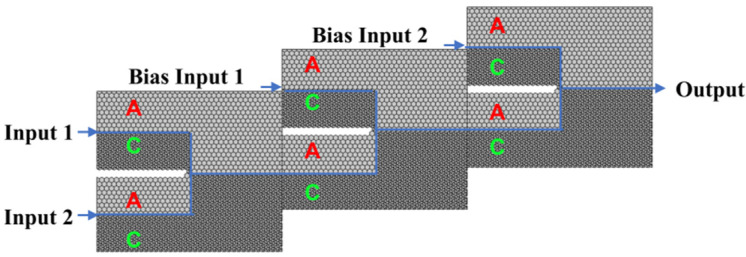
Design diagram of “AND” gate.

**Figure 11:**
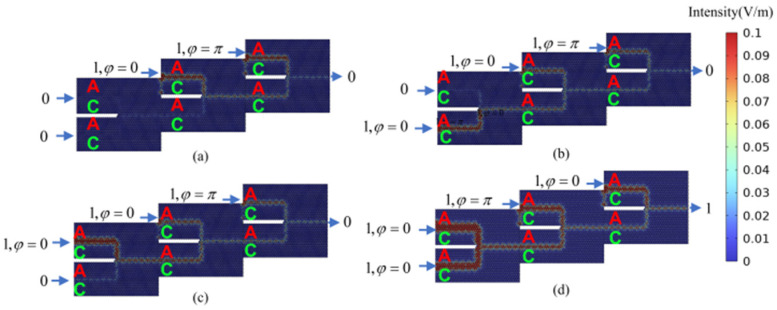
Calculation results of the light field distribution in the logic state of the “AND” gate. (**a**) “AND” gate logic input state 00, logic output state 0 (AND_00); (**b**) “AND” gate logic input state 01, logic output state 0 (AND_01); (**c**) “AND” gate logic input state 10, logic output state_ 0 (AND_10); (**d**) “AND” gate logic input state 11, logic output state 1 (AND_11).

In contrast to the previously discussed logic gates, the AND gate exhibits greater complexity, necessitating a cascade of two logic functions: NAND and NOT. The previously mentioned NAND gate is composed of two logic units, as is the NOT gate. Consequently, a direct implementation of the AND gate can be achieved by cascading four logic units. This paper proposes a simplified cascaded structure for the AND gate, utilizing only three logic units, as illustrated in Figure 10. This simplified structure comprises four input ports (input port 1, input port 2, bias light input port 1, and bias light input port 2) and a single output port. Regarding the AND gate (specifically the AND logic function applied to input port 1 and input port 2), the phase difference between input port 1 and input port 2 is maintained at Δ*φ* = 0, with the bias light activated. The AND gate logic function can be achieved by controlling the phase of the bias light signal. Figure 11a–c illustrate the cases where the logical input states of input port 1 and input port 2 are 00, 01, or 10. In these scenarios, the phase difference between bias light signal 1 and the input ports is set to Δ*φ* = 0, while the phase difference between bias light signal 2 and the input ports is set to Δ*φ* = π. This results in zero output optical signal amplitudes at the output port, which corresponds to an output logic state of 0. Figure 11d depicts the scenario where both input port 1 and input port 2 have logical input states of 11. In this instance, the phase difference between bias light signal 1 and the input ports is set to Δ*φ* = π, while the phase difference between bias light signal 2 and the input ports is set to Δ*φ* = 0. Consequently, a non-zero output optical signal amplitude is observed at the output port, indicating an output logic state of 1. Under these operational conditions, the AND gate exhibits a contrast ratio of 19.29 dB.

The calculated results obtained for the four logic states of the seven designed optical logic gates demonstrate strong agreement with the corresponding truth table (Table 1). This confirms the ability of the designed logic gates to perform logic operations, highlighting their significant potential for applications in areas such as optical computing and optical communications.

## 4. Verification of Dual-Band Computing Contrast and Robustness for All-Optical Logic Gates

**Figure 12:**
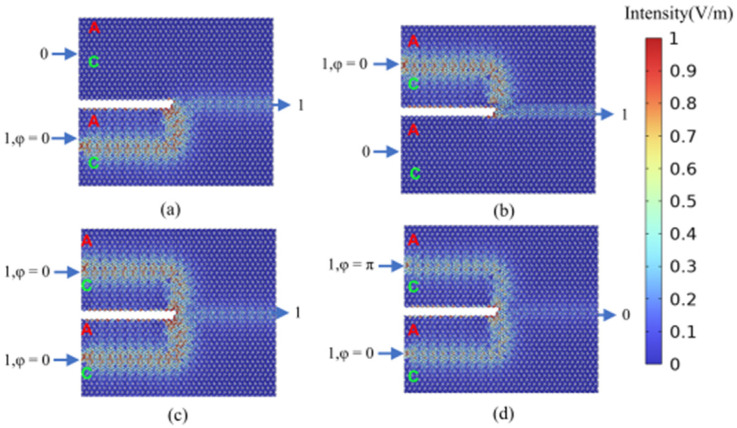
The calculation results of optical field distribution for AC-type “OR/XOR” gate logic states at 281.95 THz (1064 nm). (**a**) “OR/XOR” gate logic input state 01, logic output state 1 (OR_01(XOR_01)); (**b**) “OR/XOR” gate logic input state 10, logic output state 1 (OR_10(XOR_10)); (**c**) “OR” gate logic input state 11, logic output state 1 (OR_11); (**d**) “XOR” gate logic input state 11, logic output state 0 (XOR_11).

**Figure 13:**
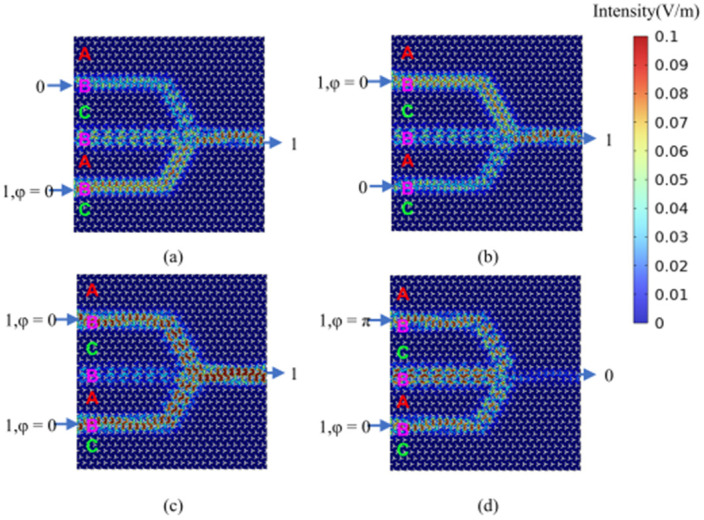
The calculation results of optical field distribution for ABC-type “OR/XOR” gate logic states at 193.54 THz (1550 nm). (**a**) “OR/XOR” gate logic input state 01, logic output state 1 (OR_01(XOR_01)); (**b**) “OR/XOR” gate logic input state 10, logic output state 1 (OR_10(XOR_10)); (**c**) “OR” gate logic input state 11, logic output state 1 (OR_11); (**d**) “XOR” gate logic input state 11, logic output state 0 (XOR_11).

**Figure 14:**
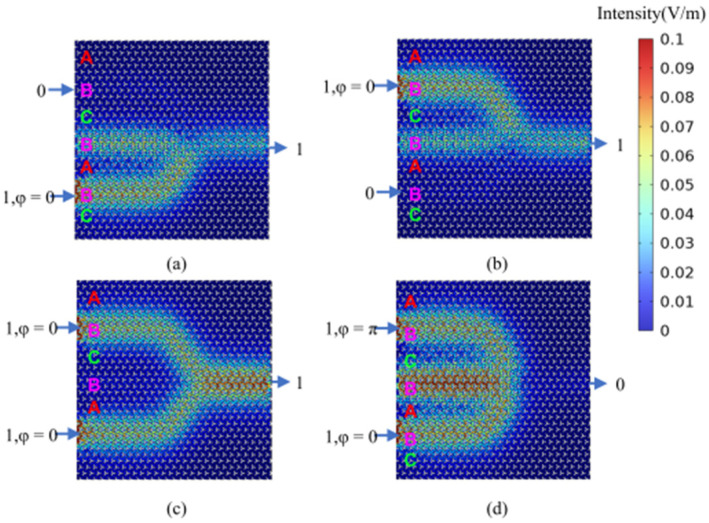
The calculation results of optical field distribution for ABC-type “OR/XOR” gate logic states at 281.95 THz (1064 nm). (**a**) “OR/XOR” gate logic input state 01, logic output state 1 (OR_01(XOR_01)); (**b**) “OR/XOR” gate logic input state 10, logic output state 1 (OR_10(XOR_10)); (**c**) “OR” gate logic input state 11, logic output state 1 (OR_11); (**d**) “XOR” gate logic input state 11, logic output state 0 (XOR_11).

The AC-type logic gates presented in this study exhibit significantly higher contrast ratios compared to conventional ABC-type logic gates. Figure 12 illustrates the calculated optical field distributions for the logic states of the AC-type OR and XOR gates at an input optical signal frequency of 281.95 THz (corresponding to a wavelength of 1064 nm). Figure 13 and Figure 14 depict the calculated optical field distributions for the logic states of the ABC-type OR and XOR gates at input optical signal frequencies of 193.54 THz (corresponding to a wavelength of 1550 nm) and 281.95 THz (corresponding to a wavelength of 1064 nm), respectively. Specifically, the contrast ratios of the ABC-type OR gates at these two wavelengths are 20.24 dB and 17.13 dB, respectively. In contrast, the AC-type OR gates demonstrate contrast ratio enhancements of 16.92 dB and 12.87 dB, respectively.

**Figure 15:**
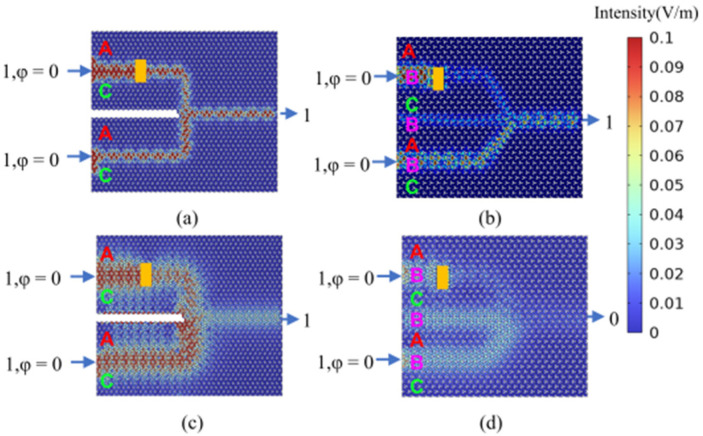
The calculation results of field distribution when introducing impurity defect in the “OR” logic gate (rectangular area represents SiO_2_ impurity defect). (**a**) Calculation results of AC-type optical field distribution at 193.54 THz (1550 nm); (**b**) calculation results of ABC-type optical field distribution at 193.54 THz (1550 nm); (**c**) calculation results of AC-type optical field distribution at 281.95 THz (1064 nm); (**d**) calculation results of ABC-type optical field distribution at 281.95 THz (1064 nm).

To assess the robustness of the proposed AC-type all-optical logic gates, impurity defects (represented by the rectangular regions in Figure 15, which simulate fabrication imperfections) were introduced into the transmission path of the OR gate. Subsequently, the optical field distribution was calculated for an input of (11). The AC-type all-optical logic gates demonstrate a significant advantage in terms of robustness compared to conventional ABC-type all-optical logic gates. Figure 15a,b present the calculated robust transmission results for the ABC-type and AC-type OR gates, respectively, at an input optical signal frequency of 193.54 THz (corresponding to a wavelength of 1550 nm). Figure 15c,d depict the calculated robust transmission results for the ABC-type and AC-type OR gates, respectively, at an input optical signal frequency of 281.95 THz (corresponding to a wavelength of 1064 nm). The results clearly indicate that even in the presence of impurity defects, the AC-type OR gate maintains significant logic output functionality.

## 5. Conclusions

Motivated by the increasing demand for all-optical computing systems that require high-bandwidth, highly integrated, low-loss, and robust all-optical logic gates, this work proposes a design methodology for AC-type dual-band logic gates based on silicon topological valley photonic crystals. Seven all-optical logic gate structures (OR, XOR, NOT, NAND, NOR, XNOR, and AND) were designed, and their functionality was verified through numerical simulations. The simulation results demonstrate that all the proposed structures successfully achieve the desired all-optical logic gate functionalities. For the designed OR gate, comparisons with the conventional ABC-type design demonstrate its superior contrast ratio in both operational wavelength bands. Furthermore, the introduction of impurity defects confirms the robust light transmission characteristics of the AC-type OR gate across both operational wavelength bands. All-optical logic gates implemented on a topological photonic crystal platform offer several key advantages, including a large operational bandwidth, a high contrast ratio, low optical loss, and robust light transmission. These characteristics can significantly enhance computational throughput, suggesting promising applications in future optical signal processing, optical communications, and optical sensing systems. Based on the theoretical design and numerical simulation verification presented in this work, future work will focus on exploring the parameter space to optimize the logic gate performance, specifically targeting the maximization of the contrast ratio. This will enable the experimental validation of the advantages offered by these logic gates in terms of high throughput, high contrast ratio, and robustness.

## Figures and Tables

**Table 1:** The truth table of optical computing logic gates.

Inputs		Logic Gates						
Input 1	Input 2	OR	XOR	NOT (Input 1)	NAND	NOR	XNOR	AND
0	0	0	0	1	1	1	1	0
0	1	1	1	1	1	0	0	0
1	0	1	1	0	1	0	0	0
1	1	1	0	0	0	0	1	1

## Data Availability

The original contributions presented in this study are included in the article; further inquiries can be directed to the corresponding author.
